# *β*_2_‐adrenergic regulation of stress hyperglycemia following hemorrhage in the obese Zucker rat

**DOI:** 10.14814/phy2.12215

**Published:** 2014-12-03

**Authors:** John S. Clemmer, Lusha Xiang, Silu Lu, Peter N. Mittwede, Robert L. Hester

**Affiliations:** 1 Department of Physiology and Biophysics, University of Mississippi Medical Center, Jackson, Mississippi

**Keywords:** Hemorrhage, hyperglycemia, obesity, *β*_2_ adrenoreceptor

## Abstract

Stress hyperglycemia following trauma has been shown to potentiate morbidity and mortality. Glucose control in obese patients can be challenging due to insulin resistance. Thus, understanding the mechanisms for glucose generation following hemorrhage may provide important insights into alternative options for glycemic control in obesity. Obesity is characterized by elevated glycogen and increased hepatic *β*_2_‐adrenergic activity, which play major roles in glucose production after hemorrhage. We hypothesized that, in obesity, hepatic glycogenolysis is enhanced during stress hyperglycemia due to increased hepatic *β*_2_‐adrenoceptor activation. Hemorrhage was performed in conscious lean Zucker (LZ) and obese Zucker rats (OZ) by withdrawing 35% total blood volume over 10 min. Liver glycogen content and plasma levels of glucose, insulin, and glucagon were measured before and 1 h after hemorrhage. The hyperglycemic response was greater in OZ as compared to LZ, but glycogen content was similarly reduced in both groups. Subsequently, OZ had a greater fall in insulin compared to LZ. Glucagon levels were significantly increased 1 h after hemorrhage in LZ but not in OZ. To test the direct adrenergic effects on the liver after hemorrhage, we treated animals before hemorrhage with a selective *β*_2_‐adrenoceptor antagonist, ICI‐118,551 (ICI; 2 mg/kg/h, i.v.). After hemorrhage, ICI significantly reduced hyperglycemia in both LZ and OZ, independent of hormonal changes, but there was a significantly decreased hepatic glycogenolysis in OZ. These results suggest that the hemorrhage‐induced hepatic glycogenolysis is likely glucagon‐dependent in LZ, whereas the *β*_2_‐adrenoceptor plays a greater role in OZ.

## Abstract (precis)

This figure demonstrates that hemorrhage does not result in an increase in glucagon levels in the obese Zucker rat, but does increase in the lean Zucker rat. These results suggest that the hemorrhage‐induced hepatic glycogenolysis is likely glucagon‐dependent in LZ and not in obese Zucker.

## Introduction

Hemorrhage is the leading cause of death after traumatic injury (Sauaia et al. 1995; Boulanger et al. 2007) with hemorrhage being the primary hyperglycemic stimulus in trauma models (Ma et al. 2003; Xu et al. 2008). Obese patients have increased morbidity and mortality after hemorrhage (Nelson et al. 2012), with impaired glucose regulation being a better predictor of increased mortality and complications than body mass index in obese patients (Sperry et al. 2007; Pieracci et al. 2008; Mowery et al. 2010). Controlling stress hyperglycemia in trauma patients has been shown to improve mortality (van den Berghe et al. 2001; Sung et al. 2005; Vogelzang et al. 2006; Egi et al. 2011), but can be challenging due to insulin resistance and increased risk of hypoglycemic episodes (Collier et al. 2008; Li and Messina 2009). Thus, understanding the mechanism for hepatic glycogenolysis in response to trauma may provide important insights into alternative options for glycemic control in obesity.

Stress hyperglycemia after hemorrhage has been documented for more than a century (Bernard 1877). During hemorrhage, there is a rapid increase in plasma glucose by an increased glycogenolysis through sympathetic activation of the hepatic *β*_2_ adrenoceptor (Arinze and Kawai 1983; Kawai and Arinze 1983) and indirectly through insulin and glucagon regulation. Sympathetic nerve activity (SNA) has been shown to regulate insulin secretion through *α*_2_‐adrenergic activation (Saito et al. 1992; Surwit et al. 1992), resulting in decreased levels of insulin after hemorrhage (Moss et al. 1972; Cerchio et al. 1973). Also, an increase in SNA during hemorrhage stimulates glucagon release primarily through both *α* and *β* adrenergic pathways (Iversen 1973; Gerich et al. 1974; Atkinson et al. 1981; Skoglund et al. 1987). These hormonal factors are key players in the hyperglycemia after hemorrhage, but their regulation in the context of obesity after hemorrhage is unknown.

Obesity is associated with an altered autonomic nervous system function which is characterized by an elevated baseline SNA (Sowers et al. 1982; Landsberg 1986; Grassi et al. 1995) and increased hepatic *β*_2_‐adrenergic activity (Begin‐Heick 1994). However, there is also evidence in obesity of a blunted ability to increase sympathetic responses to reductions in blood pressure (Schreihofer et al. 2007). A previously published paper from our group shows an impaired renin secretion after hemorrhage in obesity, suggesting an impaired renal SNA in an animal model of obesity (Xiang et al. 2013), suggesting regional differences in SNA in obesity (Vaz et al. 1997). It is unclear whether there would be an increased sympathetic‐mediated hepatic glucose output after hemorrhage in a model of obesity.

Obese Zucker rats (OZ), a widely used rat model of metabolic syndrome, are similar to obese humans with hepatic and peripheral insulin resistance, marked obesity, and dyslipidemia (Stepp et al. 2004; Xiang et al. 2005, 2008). In addition, hepatic glycogen storage, glycogenolysis, and glycogen phosphorylase activity (the rate‐limiting step in glycogenolysis) have all been reported to be elevated in human (Basu et al. 2005; Dods 2013) and animal (McCune et al. 1981; Roesler et al. 1990; Chen et al. 1993; Board et al. 1995) models of obesity. Together, these studies raise a possibility that the hepatic glycogenolysis following hemorrhage might be altered in obesity. The purpose of this paper is to examine the mechanistic differences between glycogen and hormonal regulation during hemorrhagic stress in lean Zucker rats (LZ) and OZ. We hypothesized that hepatic glycogenolysis is enhanced in obesity due to increased hepatic *β*_2_‐adrenoceptor activation. We tested this hypothesis by measuring glucose, liver glycogen content, plasma insulin, and plasma glucagon after hemorrhage with or without inhibition of the hepatic *β*_2_‐adrenoceptor.

## Methods

### Animals

Male Zucker rats (11–13 weeks) were purchased from Harlan Laboratories (Indianapolis, IN). Mean body weights for LZ were 314 ± 4 and 506 ± 9 for OZ. The experiments and protocols used in the current study were approved by the Institutional Animal Care and Use Committee at the University of Mississippi Medical Center and were carried out according to both the guidelines of the Animal Welfare Act and the Guide for the Care and Use of Laboratory Animals from the National Institutes of Health. Animals were housed 2–3 animals per cage at 22°C in a 12‐h dark/light cycle with food and water ad libitum.

### Mean arterial blood pressure

Animals were anesthetized with inhalation of 5% isoflurane with 100% oxygen. The animals' necks were shaved and wiped with 70% ethanol. The left jugular vein and right carotid artery were isolated and catheterized with polyethylene catheters (Intramedic PE‐50 tubing). Catheters contained 10% heparin in saline. Sensorcaine (0.25%) was administered on the surgical site and incisions were closed with 4‐0 Vicryl suture. Animals were allowed to recover from anesthesia for 3 h in cages with water ad libitum before the start of the experiment. Mean arterial blood pressure (MAP) was then recorded from the carotid catheter using a PowerLab system (Model ML 118; ADInstruments, Colorado Springs, CO). At the time of hemorrhage (approximately 11:00–12:00), animals had been fasting 3–4 h.

### Hemorrhage protocol

During the hemorrhage and 60‐min recovery period, animals had no access to food or water. Twenty minutes before the start of hemorrhage, baseline blood pressure was recorded and saline or the *β*_2_‐adrenoceptor antagonist, ICI 118,551 (ICI; 2 mg/kg/h i.v.), was infused via the jugular catheter (~7 *μ*L/min). Hemorrhage was initiated by withdrawing 35% total blood volume from the carotid catheter over 10 min (Xiang et al. 2013). The total blood volume calculation for the LZ was based on the linear relationship between bodyweight and blood volume: body weight × 0.06 + 0.77 (Lee and Blaufox 1985; Boku et al. 2010). Due to the lower blood volume per weight in OZ, total blood volume was calculated using the body weight of age‐matched LZ as others have published previously (Schreihofer et al. 2005; Frisbee 2006). Glucose levels were measured before and after hemorrhage using a glucometer (On Call Plus; ACON Laboratories, San Diego, CA) from blood (<5 *μ*L) collected from the tail tips pretreated with Sensorcaine. The first 2 mL of arterial blood withdrawn from the carotid catheter during the hemorrhage was collected in chilled EDTA tubes, centrifuged at 3000 *g* for 15 min, and the supernatant was stored at −80°C until the analysis of baseline plasma hormone levels. The plasma samples from animals infused with saline were used for baseline hormone levels for all groups.

### Plasma insulin, glucagon, and liver glycogen levels

One hour after hemorrhage, rats were anesthetized with isoflurane. Blood samples (2 mL) were immediately withdrawn from the carotid catheter and stored at −80°C for posthemorrhage hormone measurements. The plasma concentrations of insulin and glucagon were measured by ELISA (R&D Systems, Minneapolis, MN). Within a minute of anesthesia, the medial liver lobe was excised and immediately placed in liquid nitrogen and stored at −80°C for later measurements of glycogen using a glycogen assay kit (MAK016; Sigma–Aldrich, St. Louis, MO). Hepatic glycogen levels were normalized by liver tissue weight. Control glycogen levels were obtained from livers of LZ and OZ controls that were similarly fasted (4–5 h) but not subjected to hemorrhage.

### Statistical analysis

Absolute value changes and suppression (%) in insulin levels in LZ and OZ after hemorrhage were compared using *t*‐test. Glucose area under the curve (AUC) was calculated for the 60‐min hyperglycemia curves using the trapezoidal rule. Liver glycogen content from control animals and hemorrhage ICI‐treated animals was compared using a one‐way analysis of variance (ANOVA). All other groups were compared with two‐way ANOVA or two‐way repeated measures ANOVA test, as appropriate. Where significance was found, individual groups were compared with the Holm–Sidak post hoc test. All data are presented as mean ± standard error. A probability of *P* < 0.05 was considered as statistical significant for all comparisons.

## Results

**Figure 1.**
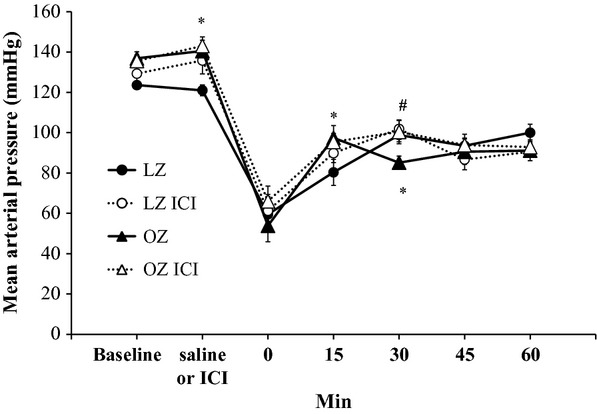
The mean arterial pressure (MAP) responses after hemorrhage in LZ and OZ with or without ICI treatment. After baseline measurements, animals were infused with saline or ICI for 20 min. Time 0 represents immediately after hemorrhage (*P < 0.05 OZ vs. LZ; ^#^*P* < 0.05 vs. OZ;* n* = 6–7 for each group).

Overall, within each group, there were no appreciable changes in MAP after 20 min of saline or ICI infusion (Fig. 1). However, after saline infusion, OZ had significantly higher MAP as compared to LZ. Hemorrhage significantly decreased MAP in all groups. After hemorrhage, OZ MAP was higher at 15 min and lower at 30 min compared to LZ. Other than a significant increase in MAP in the ICI‐treated OZ 30 min after hemorrhage, ICI treatment had no effect on MAP responses in either group.

**Figure 2.**
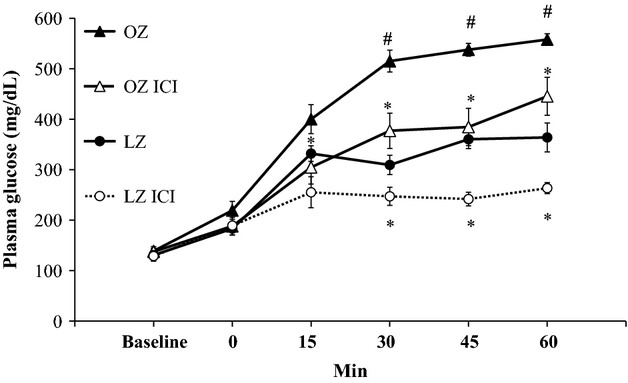
The glucose responses after hemorrhage with or without ICI treatment for 60 min after hemorrhage (Hem). Time 0 represents immediately after hemorrhage (*P < 0.05 ICI vs. control, ^#^*P* < 0.05 OZ vs. LZ;* n* = 6–7 for each group).

**Figure 3.**
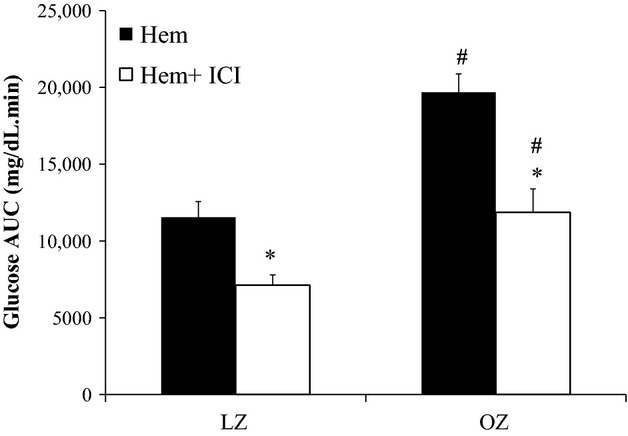
The area under the curve (AUC) for the plasma glucose responses presented in Figure 2 (*P < 0.05 vs. control, ^#^*P* < 0.05 OZ vs. LZ;* n* = 6–7 for each group).

At baseline, fasting plasma glucose was similar among all groups (Fig. 2). Compared to baseline, glucose significantly increased in all animal groups immediately after hemorrhage (0 min). OZ had significantly higher glucose levels compared to LZ starting at 30 min after hemorrhage, and this significant difference in glucose levels persisted throughout the remainder of the hemorrhage. ICI treatment significantly reduced glucose levels in both OZ (starting at 15 min) and LZ (starting at 30 min). Glucose levels remained significantly lower in ICI‐treated groups as compared to saline‐treated groups throughout the remainder of the hemorrhage. Figure 3 shows the AUC for the glucose responses. OZ had a significantly greater glucose AUC after hemorrhage compared to LZ. There was a significant fall in glucose AUC in both LZ and OZ with ICI treatment (Fig. 3). There was no major effect in the glucose‐lowering effect of ICI between LZ and OZ. However, there was a larger difference (delta change) in glucose AUC between saline and ICI‐treated OZ (7821 mg/dL/min) as compared to saline and ICI‐treated LZ (4420 mg/dL/min; Fig. 3).

**Figure 4.**
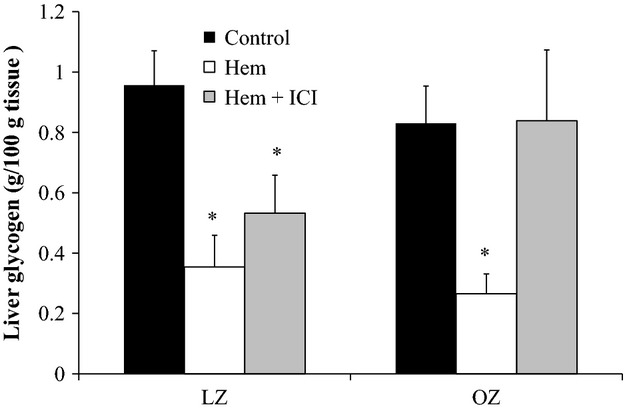
Liver glycogen normalized by tissue weight at control levels, 60 min after hemorrhage (Hem), and 60 min after hemorrhage with ICI treatment (Hem + ICI; *P < 0.05 vs. Control; *n* = 6 for all groups but ICI‐treated LZ *n* = 5).

Baseline liver glycogen levels were not different between LZ and OZ (Fig. 4). One hour after hemorrhage, there were similar reductions in glycogen content in the saline‐treated LZ and OZ. In LZ, the glycogen content tended to be higher with ICI treatment as compared to saline, however, this was not statistically significant. As compared to control, glycogen content was significantly lower 1 h after hemorrhage in ICI‐treated LZ. In contrast, in ICI‐treated OZ, glycogen content after hemorrhage was similar to control levels.

**Figure 5.**
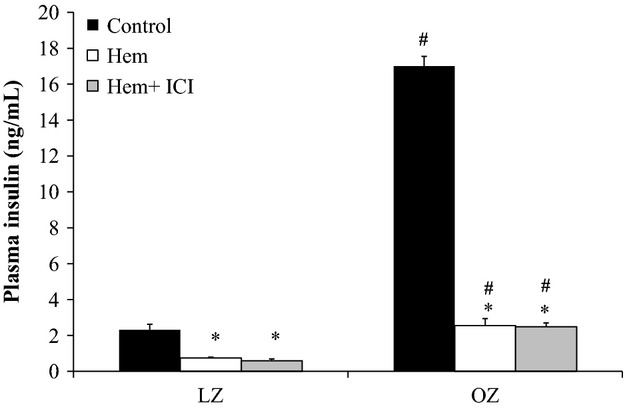
Plasma insulin levels at control levels, after hemorrhage (Hem), and after hemorrhage with ICI treatment (Hem + ICI; *P < 0.05 vs. control, ^#^*P* < 0.05 vs. LZ;* n* = 6 for all groups but ICI‐treated LZ *n* = 4).

Insulin levels were higher at baseline in OZ compared to LZ (Fig. 5). Plasma insulin decreased significantly after hemorrhage in both LZ and OZ, but OZ had an exaggerated fall in plasma insulin levels (Δ14 ± 0.8 ng/mL, corresponding to a −85 ± 2% decrease from baseline) as compared to LZ (Δ2 ± 0.3 ng/mL, corresponding to a −65 ± 4% decrease from baseline). After hemorrhage, OZ had significantly higher plasma insulin levels as compared to LZ in both saline‐ and ICI‐treated groups. There were no differences in plasma insulin in ICI‐treated animals compared to the respective saline‐treated group after hemorrhage.

**Figure 6.**
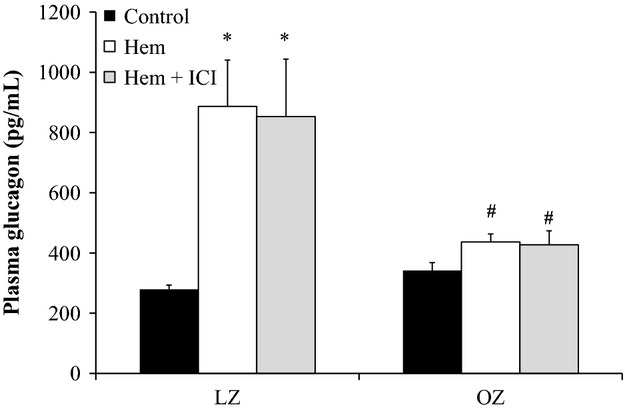
Plasma glucagon levels at control levels, after hemorrhage (Hem), and after hemorrhage with ICI treatment (Hem + ICI; *P < 0.05 vs. control, ^#^*P* < 0.05 vs. LZ;* n* = 5–6 for each group).

One hour after hemorrhage, plasma glucagon increased threefold in LZ, but there was no significant change in OZ (Fig. 6). Also after hemorrhage, OZ had significantly lower glucagon levels as compared to LZ. ICI treatment did not affect glucagon responses after hemorrhage in either group.

## Discussion

The major findings of this study are: (1) OZ, as compared to LZ, exhibited a significantly greater hyperglycemia in response to hemorrhage; (2) treatment with a *β*_2_‐adrenoceptor antagonist similarly decreased glucose levels in LZ and OZ, indicating a direct effect of sympathetic stimulation on glycogenolysis after hemorrhage; (3) ICI treatment blocked the decrease in hepatic glycogen in OZ after hemorrhage with a no significant effect in LZ, suggesting a greater *β*_2_‐adrenoceptor‐mediated glycogenolysis in OZ after hemorrhage; and (4) as compared to LZ, insulin, and glucagon levels were suppressed to a greater extent after hemorrhage in OZ and likely contributed to the altered hyperglycemic responses.

### Altered hemodynamic response in OZ after hemorrhage is insufficient to explain the exacerbated hyperglycemia

Hemorrhage can cause a rapid fall in MAP resulting in an increase in SNA. MAP was significantly reduced throughout the recovery period in all animal groups (Fig. 1). Consistent with our previous work, OZ had an impaired blood pressure recovery at only 30 min after hemorrhage (Xiang et al. 2013), with this one point in the recovery unlikely to cause significant biological differences between LZ and OZ. A reduction in blood pressure is a well‐known factor that activates the baroreflex to cause a sympathetic‐induced acute hyperglycemia. However, how this contributes to the increased plasma glucose in OZ after hemorrhage is unknown. This is complicated by the fact that OZ has impaired baroreflex sensitivity and decreased SNA in response to reductions in blood pressure (Schreihofer et al. 2007). In addition, ICI treatment had no major effects on blood pressure after hemorrhage, suggesting that peripheral *β*_2_‐adrenoceptors were not significantly activated and the improvement in posthemorrhagic hyperglycemia was independent of hemodynamic changes.

### Enhanced *β*_2_‐adrenoceptor‐mediated hepatic glycogenolysis in OZ following hemorrhage

Stress hyperglycemia occurs due to multiple pathways including the hypothalamic‐pituitary axis, inflammation, and the sympathetic nervous system. Hepatic *β*_2_‐adrenoceptor activation plays a major role in glycogenolysis (Morgan et al. 1982; Arinze and Kawai 1983). The current study reveals that the *β*_2_‐adrenoceptor is an important factor that regulates glucose levels after hemorrhage, as demonstrated by the significant reduction in the hyperglycemic responses in both LZ and OZ treated with ICI (Fig. 2). An increased *β*_2_‐adrenoceptor density and activity have been reported in isolated hepatocytes of obese mice (Begin‐Heick 1994), suggesting there could be a greater hepatic glucose output in obesity after hemorrhage due to elevated glycogenolysis through *β*_2_‐adrenoceptor activation. The AUC of the hyperglycemic responses appeared to be reduced by ICI treatment more in OZ then LZ (Fig. 3), suggesting a greater *β*_2_‐adrenoceptor‐mediated stress hyperglycemia in OZ. However, contrary to our original hypothesis, glycogenolysis did not seem to be increased in OZ after hemorrhage, as demonstrated by similarly reduced glycogen content in both LZ and OZ (Fig. 4). These data suggest that, although there were similar glycogen contents after hemorrhage, OZ, as compared to LZ, have increased *β*_2_‐adrenergic hepatic glycogenolysis.

ICI‐treated OZ had a normalization of liver glycogen content after hemorrhage, associated with a reduced hyperglycemia, suggesting a *β*_2_‐adrenoceptor‐mediated glycogenolysis. On the other hand, glycogen levels were not different between ICI‐treated LZ and LZ after hemorrhage (Fig. 4), suggesting glycogenolysis still occurred with hepatic *β*_2_‐adrenoceptor inhibition. This is most likely due to the marked increase in glucagon levels, which can dramatically increase glycogenolysis (Sokal et al. 1964; Wasserman et al. 1989).

### Increased insulin suppression in OZ following hemorrhage

The role of the sympathetic nervous system on insulin regulation is well established. The sympathetic control of insulin secretion has been shown to be primarily regulated by *α*_2_‐adrenergic activation (Saito et al. 1992; Surwit et al. 1992), resulting in decreased levels of insulin after hemorrhage (Moss et al. 1972; Cerchio et al. 1973). In the current study, *β*_2_‐adrenoceptor inhibition had no effect on insulin concentrations in either LZ or OZ after hemorrhage (Fig. 5), suggesting insulin control is independent of the *β*_2_‐adrenoceptor after hemorrhage. We also show a greater insulin suppression in OZ after hemorrhage. Our results are in agreement with a previous study that showed that, after an acute physical stress, ob/ob mice had greater glucose responses and greater insulin suppression than lean controls (Surwit et al. 1984). Also, obese patients have greater pancreatic *α*_2_‐adrenergic activity after glucose intake, which is associated with a greater suppression of insulin secretion (Robertson et al. 1976; Kashiwagi et al. 1986). It is important to note that, in the current study, although LZ and OZ had relatively similar insulin levels after hemorrhage, the low level of insulin most likely caused greater consequences in the insulin‐resistant OZ. These data suggest that after hemorrhage, obesity may be associated with increased pancreatic *α*_2_‐adrenoceptor activation resulting in decreased insulin secretion and an overall increased hyperglycemia. Blocking the *α*_2_‐adrenoceptor, however, was not feasible because there are a lack of selective pharmacological approaches that do not have unwarranted effects, including effects on pre‐ and postsynaptic *α*_2_‐adrenergic receptors that can alter synaptic norepinephrine concentrations and SNA (Jiang and Zhang 2003).

### Blunted glucagon release in OZ following hemorrhage

The role of glucagon in response to hemorrhage has not been investigated in a model of obesity. Normally, glucagon release is stimulated after hemorrhage primarily due to pancreatic SNA through nonspecific *β*‐adrenergic pathways (Gerich et al. 1974), independent of circulating catecholamines (Briand et al. 1989). ICI‐treated groups showed no changes in glucagon compared to saline controls after hemorrhage (Fig. 6), suggesting the *β*_2_‐adrenoceptor plays a minimal role in glucagon secretion in this hemorrhage model. Also, the unchanged glucagon levels after ICI treatment were associated with reduced plasma glucose levels. Sympathetic control of glucagon has been shown to override any additive effects of glucose (Bloom and Edwards 1975). This suggests that after hemorrhage, plasma glucose may play a minor role in the normal insulin and glucagon negative feedback responses. We have previously shown that there is a significant hyperglycemia after orthopedic trauma in OZ despite decreased plasma glucagon levels (Xiang et al. 2014). In the current study, the unchanged plasma glucagon in OZ after hemorrhage suggests that glucagon is not playing a major role in the acute hyperglycemia.

The mechanism for glucagon's blunted secretion in obesity after hemorrhage is unknown. Under baseline conditions, obesity is normally associated with elevated plasma glucagon and pancreatic *α* cell activity (Unger et al. 1970; Reach and Assan 1979). However, under stress, obesity may cause alterations in glucagon regulation. Glucagon secretion can be sympathetically inhibited through *α*‐adrenergic mechanisms (Saito et al. 1992; Surwit et al. 1992), which may be due to a similar mechanism as the suppressed insulin release after hemorrhage in OZ. Although there is minimal evidence on endocrine regulation in obesity after hemorrhage, there is ample data on obesity and exercise, another situation when SNA is elevated and insulin secretion is suppressed. Indeed, glucagon increases and insulin tends to fall during exercise (Bielinski et al. 1985; Wolfe et al. 1986; Marliss and Vranic 2002). However, obese patients have a blunted glucagon secretion after exercise (Gustafson et al. 1990). Moreover, sympathetic‐mediated increases in glucagon secretion have been shown to be diminished in diabetic patients (Shamoon et al. 1980). These data suggest that obesity may alter glucagon responses to acute hemorrhage. Novel glucagon receptor antagonists are currently being investigated in clinical trials (Petersen and Sullivan 2001; Christensen et al. 2011; Engel et al. 2011a,b) and could theoretically be used in the future to control acute hyperglycemia after trauma and hemorrhage. The data from the current study suggest that this may not be an effective strategy in the obese patient.

### Limitations

Although the current study found similar fasting plasma glucose levels between LZ and OZ, OZ are characterized by both hepatic and peripheral insulin resistance. While the current study's focus was on glucose generation and possible mechanisms, the data do not account for baseline or secondary insulin resistance that can occur after hemorrhage and trauma. This may account for the similar magnitude of ICI treatment to lower glucose levels in LZ and OZ. The current data suggest OZ to have higher *β*_2_‐adrenergic‐mediated glycogenolysis, yet, the presence of an altered baseline or secondary resistance to insulin after hemorrhage could have effected glucose disposal and promote higher glucose values in the ICI‐treated OZ.

There was a significant difference found in the effect of ICI on liver glycogen content between LZ and OZ, suggesting exaggerated *β*_2_‐mediated glycogenolysis. However, the effect of ICI treatment on plasma glucose seemed to be similar between LZ and OZ. This suggests that other factors such as muscle glycogen, muscle glucose uptake, and insulin's effect on these may play a role in the resultant plasma glucose levels after hemorrhage.

### Conclusion

As compared to LZ, OZ exhibited increased hyperglycemia after hemorrhage, which may be due to impaired glucose uptake due to a greater insulin suppression and/or baseline insulin resistance in OZ, but not glucagon secretion. Future studies are needed to determine if obesity plays a role in altering pancreatic *α*_2_‐adrenoceptor activation after hemorrhage, resulting in exaggerated suppression of both insulin and glucagon secretion. Also, these findings suggest obesity is associated with increased hepatic *β*_2_‐adrenergic glycogenolysis after hemorrhage. The current data provide important clues for controlling glucose after hemorrhage in the context of obesity.

## Acknowledgments

We thank H. Zhang for performing the hormonal analysis. We also thank T. E. Lohmeier for his comments in the review of the manuscript.

## Conflict of Interest

None declared.
